# Editorial: Vascular pathophysiology in hypoxia

**DOI:** 10.3389/fphys.2023.1235383

**Published:** 2023-07-07

**Authors:** Kusal K. Das, Dewan S. A. Majid, Nanduri R. Prabhakar

**Affiliations:** ^1^ Laboratory of Vascular Physiology and Medicine, Department of Physiology, Shri B. M. Patil Medical College, Hospital and Research Centre, BLDE (Deemed to be University), Vijayapur, Karnataka, India; ^2^ Department of Physiology, Hypertension & Renal Center of Excellence, Tulane University School of Medicine, New Orleans, LA, United States; ^3^ Department of Medicine, Biological Sciences Division, Institute for Integrative Physiology, Centre for Systems Biology of Oxygen Sensing, University of Chicago, Chicago, IL, United States

**Keywords:** hypoxia, arterial smooth muscle, hypertension, stroke, respiratory diseases

An adequate supply of O_2_ is essential for mammalian survival. Hypoxia (i.e., reduced O_2_ availability) occurs under a variety of physiological and pathological situations with profound impact on physiological systems. The duration of hypoxia can be brief lasting several seconds to minutes or can be chronic lasting several hours to days such as that encountered at high altitude sojourn. How homeostasis is maintained during hypoxia continues to be an important question of many ongoing investigations in physiology.

Acute hypoxia increases sympathetic tone, blood pressure and breathing within seconds after its onset. These rapid systemic responses to hypoxia are reflex in nature triggered by the carotid bodies, which are major sensory receptors for detecting O_2_ levels in arterial blood. On the other hand, chronic hypoxia such as experienced at high altitude maintains homeostasis through transcriptional activation of genes by hypoxia-inducible factors (HIFs) including HIF-1 and HIF-2. HIF-mediated activation of the Epo gene improves O_2_ carrying capacity by increasing erythropoietin protein. Chronic hypoxia increases formation of new blood vessels (angiogenesis) through HIF-dependent activation of vascular endothelial factor (VEGF).

Unlike individuals living at high altitude, most people living at sea level encounter chronic intermittent hypoxia (CIH) because of sleep-disorder breathing manifested as obstructive sleep apnoea (OSA). Unlike continuous hypoxia, which increases both HIF-1 and HIF-2, CIH leads to imbalanced expression of HIF-1 and HIF-2, leading to increased oxidative stress and cardio-respiratory pathophysiology.

Hypoxia can also act directly on the vasculature leading to disturbed vascular homeostasis. However, the effect of hypoxia depends on the blood vessel type. Hypoxia dilates large blood vessels either due to direct effects of low O_2_ on vascular smooth muscle and/or indirectly increasing vasodilator metabolites. In contrast, hypoxia constricts microvasculature by acting on endothelium. Hypoxic pulmonary vasoconstriction (HPV) and the resulting pulmonary hypertension represent a well-documented effects of hypoxia on microvasculature. Increased vascular permeability arising from direct effect of hypoxia on microvasculature has dire physiological consequences.

The Research Topic of Frontiers in Physiology focuses on articles addressing mechanisms underlying vascular pathologies of hypoxia. The article by Raghavan et al. address the relationship between purinergic signaling in altered vascular permeability by hypoxia. They reported that endothelial cell P2Y1 receptors mediate hypoxia-evoked endothelial barrier dysfunction and hyperpermeability, and these effects are prevented by P2Y1R antagonist. They further showed *in vitro* hypoxia/reoxygenation upregulates endothelial cell P2Y1receptors, which contributes to degradation of endothelial junctional proteins resulting in increased endothelial permeability. MRS 2500, a P2Y1R antagonist, inhibit P2Y1 receptors and improved endothelial barrier permeability. Malkmus et al. assessed the roles of Ca^2+^ regulated transient receptor potential (TRPC) proteins and pulmonary vascular remodeling in chronic hypoxia-induced pulmonary hypertension (CHPH). They hypothesized altered [Ca^2+^]_I_ as one of the mediators of pulmonary vascular remodeling and assessed whether genetic deletion of TRPC 1,3,6 channels protect against CHPH in a murine model. Their findings indicate deletion of TRPC1, 3 and 6 partially protect against CHPH without affecting pulmonary vascular remodeling. Parvatikar et al. addressed efficacies of bioactive compounds from a medicinal plant (Mucuna pruriens) on cerebral ischemia and pathophysiology of brain tissue. Their work demonstrated Mucuna prureins plant extract exhibit neuroprotective actions involving downregulation of the NMDAR and tau proteins. They identified β-sitosterol as an active compound of Mucuna prureins. Lade et al. assessed the mechanisms of hypoxia induced interactions between Na^+^/H^+^ exchanger isoform 1 (NHE1) and actin filament (via p-ezrin) in pulmonary artery smooth muscle cell (PASMC). Their findings showed hypoxia increases p-ezrin and NHE1 proteins facilitating changes in PASMC phenotype and promoting vascular remodeling and develop pulmonary hypertension. Müller et al. presented OSA-related model of intermittent hypoxia (IH) in endothelial cells and its relation to vascular pathology. Their study based on patient data with OSA provides insights into inflammatory endothelial cell activation by IH which may facilitate understanding of therapeutic aspects of IH mediated vascular pathology. Moreno-Domínguez et al. reviewed the nature of oxygen sensing in acute vasomotor response to hypoxia. They discussed two classic vasomotor responses to hypoxia including hypoxic pulmonary vasoconstriction (HPV) and hypoxic vasodilation (HVD). The review provides important translational perspectives of cardiorespiratory pathophysiology and pharmacology.

